# Airborne Cyanobacterial Toxins and Their Links to Neurodegenerative Diseases

**DOI:** 10.3390/molecules30112320

**Published:** 2025-05-26

**Authors:** Zachary James Morris, Elijah W. Stommel, James Spencer Metcalf

**Affiliations:** 1 Department of Biological Sciences, Bowling Green State University, Bowling Green, OH 43403, USA; jmetcal@bgsu.edu; 2 Department of Neurology, Geisel School of Medicine at Dartmouth, Dartmouth-Hitchcock Medical Center, Lebanon, NH 03756, USA; elijah.w.stommel@hitchcock.org; 3 Brain Chemistry Labs, Jackson, WY 83001, USA

**Keywords:** cyanobacteria, cyanotoxin, microcystin, beta-N-methylamino-L-alanine (BMAA), 2,4-diaminobutyric acid (DAB), N-(2-aminoethyl)glycine (AEG), saxitoxin, cylindrospermopsin, anatoxin-a, guanitoxin

## Abstract

Cyanobacteria can produce a wide range of toxins which have acute and chronic adverse health effects. Affecting a variety of mammalian systems, they are generally characterized according to their mode of action and the organs affected. Cyanobacterial neurotoxins are one cyanotoxin class that can negatively affect human health, and representatives of other cyanotoxins classes are increasingly showing neurotoxic effects. Of the various human exposure routes to cyanobacterial toxins, the significance of the airborne and inhalation route requires much greater clarity and understanding. People may be exposed to mixtures of cyanobacterial neurotoxins through the inhalation of sprays and dust, along with the potential to directly enter the central nervous system when crossing the blood-brain barrier. This review aims to summarize the current state of knowledge concerning airborne cyanobacterial neurotoxins, research gaps, health effects, and the need for management practices to protect human and animal health.

## 1. Introduction

Cyanobacteria are a diverse bacterial phylum which emerged ~3.5 billion years ago [1]. Thought to be responsible for oxygenating the early Earth atmosphere, cyanobacteria helped establish the conditions necessary for the evolution of the current biosphere. Cyanobacteria inhabit a wide range of aquatic and terrestrial habitats and can tolerate varied environmental conditions including hot and cold deserts, oceans, lakes, soil, and thermal features such as hot springs [2,3]. Many cyanobacteria exist as vegetative cells that are metabolically active and capable of reproduction, whereas some genera form differentiated cells such as heterocysts which specialize in nitrogen fixation [4].

Cyanobacteria are keystone organisms in most ecosystems as evidenced by their environmental ubiquity [3,5] and have adapted well to anthropogenic changes to the environment resulting from climate change and pollution [6,7,8,9]. Concerning climate change, increasing average global temperatures are likely to favor the growth of cyanobacteria over other photoautotrophic organisms [8,10]. Similarly, nutrient pollution has also increased [9,11,12], with nitrogen and phosphorous enrichment resulting in eutrophication, and permitting massive cyanobacterial blooms to form, sometimes reaching hundreds of square kilometers in area, such as in Lake Erie [13,14,15]. Often unsightly when environmental conditions deteriorate and blooms break down, cyanobacterial die-offs can lead to adverse water quality issues when bloom material is biologically degraded [16]. As cyanobacterial blooms grow and become more dense, toxic substances synthesized by cyanobacteria become more concentrated [17]. Through observations of wild and domestic animals (such as livestock), associations of cyanobacterial blooms with mass animal deaths have been derived [18]. These deaths are the result of small molecular weight secondary metabolites, known as cyanotoxins, which have adverse short and long-term health implications [19,20,21,22,23].

Many cyanobacteria are capable of synthesizing cyanotoxins which may vary in terms of chemical structure, mechanisms of toxicity, and subsequent adverse health conditions resulting from exposure. Of growing concern are cyanotoxins associated with neurodegenerative conditions such as Amyotrophic Lateral Sclerosis (ALS), Parkinson’s Disease (PD), and various forms of dementia such as Alzheimer’s Disease (AD). Cyanobacteria may synthesize toxins in response to variations in pH, temperature, light intensity, and available nitrogen and phosphorus [24,25,26,27,28,29], in addition to their potential roles as a defense mechanism against grazers or competitors. Exposure to neurotoxicologically relevant cyanotoxins may occur through various exposure routes including food [30,31,32,33,34,35], dietary supplements [36,37], drinking water [28,38], maternal transfer [39,40,41], and inhalation [42,43]. Of these exposure routes, exposure via inhalation is less studied, yet one of the most relevant, as cyanobacteria and cyanotoxins are likely to be easily transported through the atmosphere. As a result, humans and wildlife living near sites of harmful cyanobacterial blooms may be easily and frequently exposed. The purpose of this review is to outline the prominent cyanotoxins associated with neurological impairment and discuss atmospheric transfer and subsequent inhalation as an exposure route, in addition to mitigation strategies to prevent such exposures.

## 2. Cyanobacterial Neurotoxins

Cyanobacteria synthesize many secondary metabolites which are harmful to humans and wildlife. Several of these secondary metabolites have been studied extensively due to their potency and environmental relevance. This section considers cyanotoxins which may be the most harmful to human health from a neurological perspective, with deleterious effects observed in neuronal and non-neuronal systems (Table 1).

### 2.1. Anatoxin-a

**Figure 1:**
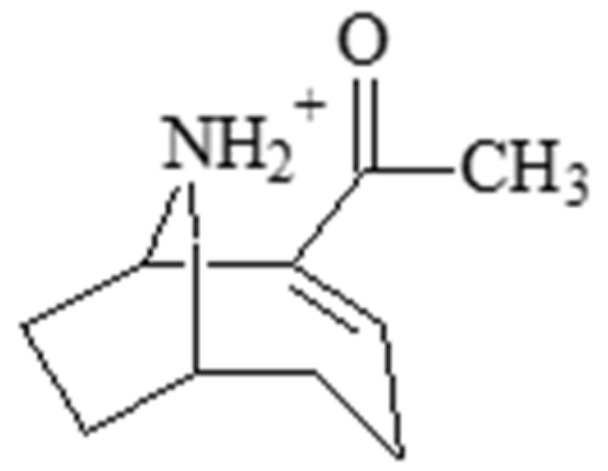
Structure of anatoxin-a.

The alkaloid cyanotoxin anatoxin-a (Figure 1) is synthesized by cyanobacterial genera including *Anabaena*, *Lyngbya*, *Oscillatoria*, *Planktothrix*, *Raphidiopsis*, and *Woronichinia* [91,92,93,94]. Previously called “very fast death factor”, anatoxin-a was characterized as a depolarizing agent in neurological tissues from multiple organisms [91,92,93,94] and bears some structural similarity to neurological stimulants such as cocaine [44,95]. Synthesis of anatoxin-a and variants may be positively correlated with environmental nitrogen and phosphorous concentrations [96,97,98], although some studies show that anatoxin-a synthesis may peak with moderate nitrogen stress [99]. Anatoxin-a mimics acetylcholine, a stimulatory neurotransmitter which interacts mainly with nicotinic acetylcholine receptors [44,45]. As acetylcholinesterase is unable to degrade anatoxin-a, neuronal stimulation cannot be attenuated, resulting in overstimulation [44]. In sufficient doses, exposure to anatoxin-a can result in paralysis, asphyxiation, abnormal muscular contraction, and death. Anatoxin-a is thought to be responsible for the deaths of animals such as domestic dogs and aquatic birds [100,101]. Apart from the known neurological effects, anatoxin-a is thought to induce cell death by apoptosis in rat thymocytes and fish lymphocytes, which may be caused by elevated concentrations of reactive oxygen species (ROS) upon exposure [46,47]. Fish lymphocytes exposed to 0.01 mg/L anatoxin-a also displayed significantly lower concentrations of enzymes which alleviate ROS stress, such as superoxide dismutase (SOD), catalase, glutathione reductase (GR) and glutathione peroxidase (GP) [47].

### 2.2. Guanitoxin

**Figure 2:**
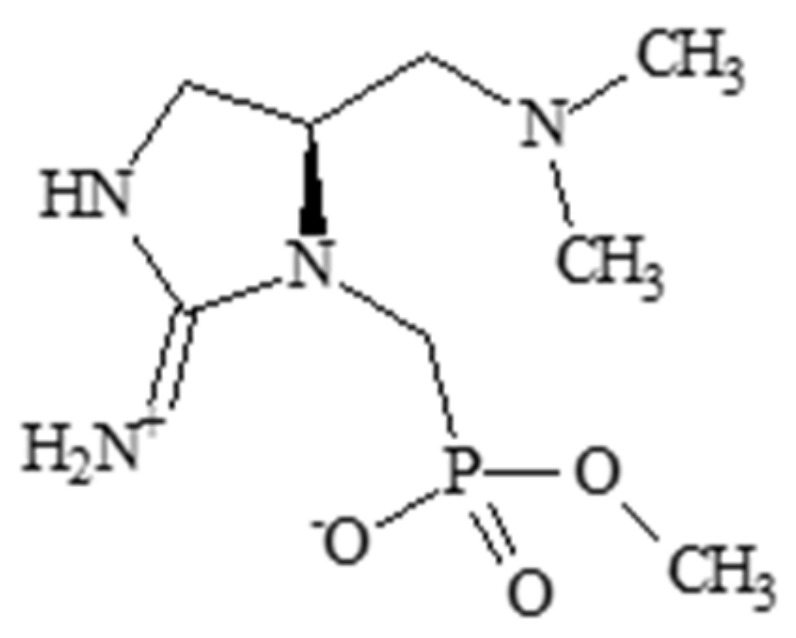
Structure of guanitoxin.

Guanitoxin (Figure 2) is an organophosphate synthesized by the cyanobacterial genera *Dolichospermum* (*Anabaena*), *Sphaerospermopsis*, *Aphanizomenon*, *Cuspidothrix* and possibly other genera such as *Microcoleus* [102,103,104]. Comparing the toxicity of guanitoxin with anatoxin-a revealed similar toxicological effects in a variety of tissues, in addition to profuse salivation in test organisms [44,92,102]. Formerly known as anatoxin-a (S) (“S” denoting salivation), the nomenclature was changed to “guanitoxin” due to significant differences from anatoxin-a in terms of structure, mechanism of toxicity, and lethal dose [48]. Unlike anatoxin-a, which mimics acetylcholine, guanitoxin prevents acetylcholine degradation by inhibiting acetylcholinesterase [102]. This inhibition can result in neurotoxicity by overstimulation, manifesting as paralysis, asphyxiation, and death. Although relatively little is known regarding the toxic effects of guanitoxin apart from inhibition of acetylcholinesterase, fish exposed to guanitoxin-producing cyanobacteria displayed changes in ROS-associated enzymes such as SOD, CAT, and glutathione-S-transferase (GST), the presence of micronuclei, and osmoregulatory disorders [49].

### 2.3. Saxitoxin

**Figure 3:**
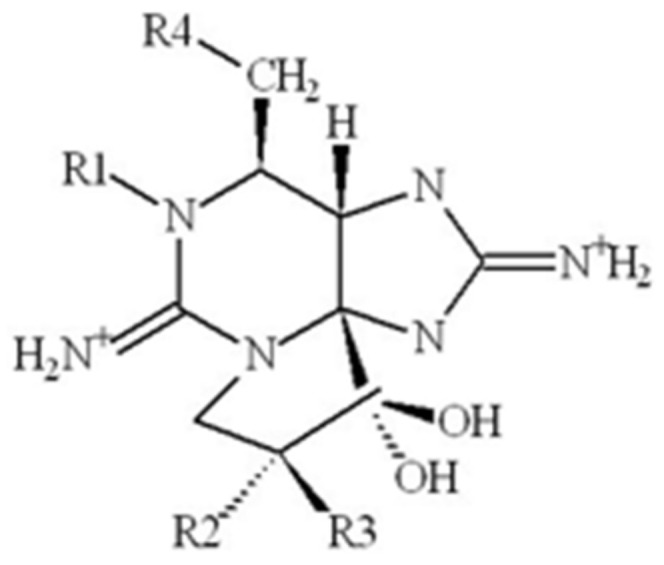
General structure of saxitoxin.

Saxitoxins (Figure 3) are synthesized by cyanobacterial genera such as *Lyngbya*, *Aphanizomenon*, *Dolichospermum*, *Planktothrix*, and *Cylindrospermopsis* [105,106]. The general structure consists of trialkyl tetrahydropurines with variable regions throughout the molecule [50,51]. Saxitoxins can be classified as C and G toxins, as well as the LW toxins produced by *L. wollei* [52]. Saxitoxins can block voltage-gated ion channels and prevent neural signaling [50,52]. The main target of saxitoxins are voltage-gated sodium channels, which take part in action potential generation. However, some variants of saxitoxin can block voltage-gated potassium and calcium channels, as well as nitric oxide synthases [50]. Toxic to many species, saxitoxins synthesized in marine environments are known to accumulate in shellfish and are the cause of paralytic shellfish poisoning [106,107,108]. Saxitoxins may also enact neurotoxicity through oxidative stress. Various organisms including rats, fish, *Caenorhabditis elegans*, and *Daphnia magna* show altered expression of enzymes such as catalase and synthesis of metabolites such as glutathione in neurological tissue when exposed to saxitoxins [53,54,55,109].

### 2.4. Neurotoxic Amino Acids

**Figure 4:**
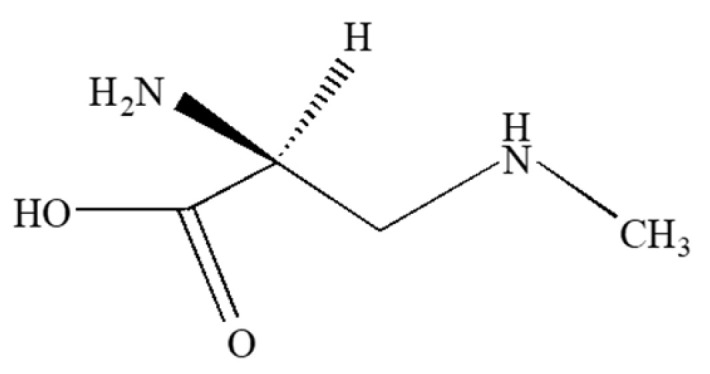
Structure of BMAA.

Neurotoxic amino acids (Figure 4) such as β-*N*-methylamino-L-alanine (BMAA), N-(2-aminoethyl)-glycine (AEG) and 2,4-diaminobutyric acid (DAB) are thought to be synthesized by most, if not all, cyanobacterial genera [30,110,111,112,113,114]. The synthesis of BMAA, AEG, and DAB may be linked to concentrations of available nitrogen, phosphorus, light, and pH [24,25,26,27,29]. BMAA can bioaccumulate through trophic levels in nature [30,31,32,33,115] and may accumulate in various regions of the brain in humans and other mammals [30,31,116]. In the eukaryotic cell, BMAA competes with the amino acid L-serine for misincorporation into proteins [56,57] and may cause protein misfolding and aggregation [116]. BMAA may also cause excitotoxicity by interacting with glutamate receptors [58,59,117,118] and is thought to selectively damage motor neurons [59,119,120]. Symptoms of ALS, PD, and dementia are associated with chronic exposure to BMAA in humans and non-human primates [30,56,121]. BMAA may also affect the proliferation of neural cancer cells, albeit with different effects observed depending on the cell line [122].

Some studies have shown that both DAB and AEG are more neurotoxic than BMAA, although toxicity can vary depending on the test organism and choice of bioassay [59,123,124,125]. AEG and DAB may be more neurotoxic than BMAA when tested in cortical cultures [59]. Although the exact mechanisms of AEG toxicity are not clear, DAB has been shown to be more excitotoxic than BMAA in the nervous systems of some invertebrates [125].

Exposure to neurotoxic amino acids may also induce toxicity by oxidative stress, epigenetic changes, impaired neurite development, apoptosis, neuronal inflammation, and morphological changes in neuronal mitochondria [59,60,61,62,63,64]. Like other toxins, BMAA is associated with elevated concentrations of enzymes associated with oxidative stress observed in some organisms, and elevated concentrations of ROS as well as DNA damage in neural stem cells [60,62]. BMAA and AEG can inhibit the transport of the glutathione precursor cystine into primary neuronal cultures [59]. Neuroinflammatory biomarkers such as elevated levels of COX2, nuclear NF-kB, TNF-alpha, and IL-1 beta expression are also associated with BMAA exposure. Finally, consistent with its role as a causative agent of ALS, exposure to BMAA is correlated with the presence of TDP-43 aggregates [63,64].

### 2.5. Microcystin

**Figure 5:**
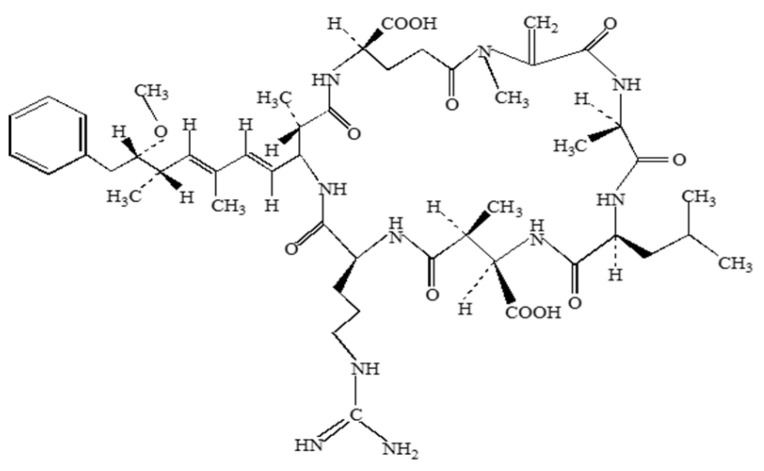
Structure of Microcystin-LR (MC-LR).

Microcystins are cyclic heptapeptides synthesized by cyanobacterial genera including *Microcystis*, *Planktothrix*, and *Nostoc* [126,127]. Over 240 microcystin congeners have been identified, due largely to amino acid substitutions, including at two positions in the peptide structure, “X” and “Z”, giving rise to the nomenclature of these toxins [128]. Microcystin synthesis is correlated with concentrations of available nitrogen and phosphorous [29,129]. Microcystins are hepatotoxins, inhibitors of phosphatases such as PP2A and tumor promoters [130,131,132]. Several mechanisms exist by which microcystin elicit toxicity. Microcystin-LR (MC-LR) (Figure 5) can cause ROS stress as evidenced by the dose dependent elevation of hydrogen peroxide in various cell types as well as altered expression of ROS-associated enzymes [65,66,67,68,69]. MC-LR can also induce neuroinflammation, as changes in expression of proinflammatory cytokines such as tumor necrosis factor alpha (TNF-alpha), interleukin-1 beta (IL-1 beta) and interleukin-6 (IL-6), along with enzymes such as nitric oxide synthase (NOS) and COX-2 have all been associated with MC-LR exposure [70,71,72,73,74,75]. The ability of microcystin to inhibit PP2A, whose function is necessary for proper neuronal function, could explain some neurological symptoms associated with MC exposure [133,134,135]. Chronic exposure to MC-LR can lead to disruption of the blood brain barrier (BBB) and neuroinflammation [71,72,136]. Microcystin effects on the BBB are likely brought on by disruption of tight junctions, increasing expression of matrix metalo-proteases (MMPs) and low levels of tight junction proteins such as Occludin and Claudin 5 [72]. Microcystins have also been shown to alter neuronal signaling in *Aplysia* and rats, albeit by different mechanisms. In *Aplysia*, MC-LR may prolong inward ion currents induced by serotonin and cyclic adenosine monophosphate [76], while in the rat hippocampal dentate gyrus, MC-LR inhibits long-term potentiation (LTP), leading to inhibition of spatial memory [77]. In rats, MC-LR may be responsible for the formation of Lewy bodies and cell-to-cell transfer of alpha-synuclein in the nervous system [71].

### 2.6. Cylindrospermopsin

**Figure 6:**
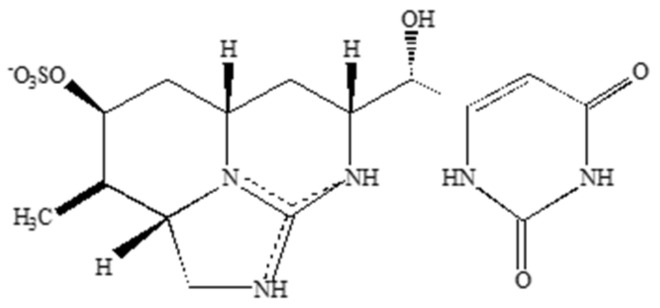
Structure of Cylindrospermopsin.

Cylindrospermopsin (Figure 6) is an alkaloid cyanotoxin, consisting of a tricyclic guanidine moiety and a hydroxymethyluracil [137]. Synthesized by several cyanobacterial genera including *Cylindrospermopsis*, *Umezakia*, *Aphanizomenon*, *Raphidiopsis*, and *Anabaena* [78], cylindrospermopsin is of growing concern due to its potent cytotoxicity. One mechanism of cylindrospermopsin toxicity, translation inhibition, may lead to the buildup of reactive oxygen species (ROS), as free radical scavengers such as glutathione may not be synthesized, potentially leading to cell death by apoptosis and DNA damage [79,80,81,82,83,84,85]. It should be noted that the toxic effects of cylindrospermopsin may change depending on cell type [22,138,139,140,141,142]. While cylindrospermopsin has deleterious effects on multiple cell types including neurons [78], less is known regarding potential mechanisms of neuronal toxicity. Hinojosa et al. showed decreased viability in murine primary neuronal cultures exposed to cylindrospermopsin and proposed that cylindrospermopsin may disrupt synaptic integrity [86]. Cylindrospermopsin can also cross the BBB and disrupt acetylcholine activity in certain fish species [87,88,89]. Some evidence shows that cylindrospermopsin can cause neuroinflammation in BV-2 and N2a cells with elevated levels of TNF-alpha in each upon exposure [90].

## 3. Cyanotoxin Exposure Routes

### 3.1. Exposure to Cyanotoxins from Aquatic Environments

Globally, cyanotoxins have been implicated in intoxications of humans and wildlife. In 2014, roughly 500,000 Ohioans living in the Toledo area were without potable water due to the release of microcystins such as MC-LR from a large cyanobacterial bloom in the Western Basin of Lake Erie [28,38,143]. Animals which use cyanotoxin-contaminated water for drinking or as a habitat are also at risk of intoxication. For example, cyanotoxin exposure was thought to be responsible for the deaths of African elephants that drank from water sources known for cyanobacterial blooms [144]. Guanitoxin has been noted as the cause of waterfowl poisonings in Danish Lakes [52,145], and the deaths of livestock have been reported after consumption of water containing cylindrospermopsin [22]. Cyanotoxins may also be responsible for marine cetacean deaths [146].

Edible marine invertebrates such as shrimp and mollusks can accumulate BMAA in their tissues [33,147], and saxitoxins in shellfish may lead to shellfish poisoning [106,108]. Fish tissues can accumulate cyanotoxins in both marine and freshwater environments. BMAA was detected in the tissues of a variety of edible fish species in the Black Sea, as well as in carp from Lake Mascoma, New Hampshire amongst others [115,147,148,149]. More recently, BMAA has been detected in Lake Erie fish, which are consumed after being caught recreationally and commercially [150].

Cyanotoxins originating from aquatic environments may also impact terrestrial environments, albeit indirectly, as water contaminated with cyanotoxins may be used in agriculture. As cyanotoxins can accumulate in a variety of plants, exposure may occur through the consumption of contaminated plant matter after irrigation with cyanotoxin-containing water [30,151,152,153,154,155,156,157,158,159,160]. Mohammad et al. showed that BMAA contaminated water used to irrigate edible plants accumulated to detectable levels in both free and protein-associated states [158]. Microcystin can accumulate in edible plants such as lettuces, radishes, and carrots after irrigation with *Microcystis* and/or microcystin-containing water, which may hinder plant development depending on the concentration and duration of exposure [155]. The use of aquatic organisms for commercial animal feed can also lead to accumulation of BMAA in livestock [33].

### 3.2. Exposure to Cyanotoxins from Terrestrial Environments

Terrestrial cyanobacteria are capable of synthesizing many of the same toxins as aquatic species. On Guam, an unusually high number of individuals showed symptoms of Amyotrophic Lateral Sclerosis/Parkinsonism Dementia complex (ALS/PDC) which includes characteristic symptoms of ALS, AD, and PD [161,162]. Cox et al. examined *Nostoc* species inhabiting the coralloid roots of the cycad *Cycas micronesica* which were found to produce BMAA. BMAA was present at high concentrations in the seeds of the cycad which were consumed by flying foxes native to Guam. BMAA in the flying foxes and cycad seeds accumulated >1000-fold compared to that detected in the *Nostoc* associated with the cycad coralloid root. Cycad flour prepared by native Chamorro people also contained free and protein bound BMAA. Those who consumed flying foxes and cycad seed flour over extended periods of time accumulated BMAA in brain tissues [30,31].

Another possible exposure route to terrestrial cyanotoxins are biological soil crusts. Biological soil crusts are important in arid environments where they stabilize the uppermost soil surfaces, binding soil particles together and providing a physical barrier preventing erosion [163,164]. As cyanobacteria may constitute a large proportion of the biomass found in soil crusts [103,124,165,166,167,168], cyanotoxins have the potential to occur at varying concentrations and types depending on environmental conditions and the composition of genera present [167]. In the desert soils of Qatar, for example, prominent cyanobacterial genera included *Microcoleus*, *Phormidium*, and *Chroococcus* [166,168,169] and cyanotoxins such as microcystin, guanitoxin, BMAA, DAB, and AEG have been found in crust material [103]. In the Arctic, microcystin and anatoxin-a have been detected in biological soil crusts [170]. Biological soil crusts from loess deposits in Iran showed great diversity of cyanobacterial genera, but varying toxicity [167].

Other terrestrial sources of cyanotoxins include dry lake beds and cyanobacteria found in arctic regions. Dry and receding lake beds such as the Great Salt Lake in the State of Utah are thought to be sources of airborne BMAA, AEG, and DAB [171]. One final source of terrestrial cyanotoxins comes from cyanobacterial mats, such as those found in the Antarctic from which BMAA and microcystins were detected [172].

### 3.3. Exposure to Cyanotoxins from Food and Dietary Supplements

Dietary supplements containing or consisting entirely of cyanobacteria are popular due to their high nutrient content. Species of the genus *Arthrospira* are consumed in Central Africa [173] and are marketed in the United States along with *Aphanizomenon flos-aquae* as the dietary supplement “spirulina”. Despite being considered non-toxic, representatives of these cyanobacteria can synthesize a variety of toxins or may be contaminated with toxic cyanobacterial species before processing for consumption. In a screen for 37 cyanotoxins in supplements which included spirulina and *Aphanizomenon flos-aquae*, Fontaine et al. detected microcystins, DAB, anatoxin-a, and beta-amino-N-methylamine (BAMA) [35]. Roy-Lachapelle et al. also screened dietary supplements for cyanotoxins, with several detected above levels considered tolerable for daily intake [36]. However, as certain toxins such as BMAA have the potential to bioaccumulate in human tissues [30,31], repeated consumption of dietary supplements containing cyanobacteria may serve as a route of chronic exposure and potentially lead to neurological conditions. Metcalf et al. examined *Arthrospira* consumed by people in the African nation of Chad and provided evidence for the presence of microcystins and DAB [173]. *Nostoc* species such as *Nostoc commune* and *Nostoc flagelliforme* are consumed whole or as fa cai noodles in Peru and China respectively, and have both been shown to contain BMAA [34,174].

### 3.4. Exposure to Cyanotoxins in the Atmosphere

Although exposure to cyanotoxins from aquatic and terrestrial environments, as well as through food and dietary supplements, is well documented, exposure to cyanobacteria and cyanotoxins via the atmosphere is less well studied. Cyanobacteria may constitute a significant proportion of aerial microbial communities, as they can withstand a range of environmental conditions [169,175,176,177,178,179,180,181]. This presents unique health risks, as air currents can carry particles containing cyanobacteria and cyanotoxins over long distances [182], potentially being able to adversely affect individuals over broad spatial distributions. Cyanobacteria have also been detected in the atmosphere of indoor facilities, implying that air filtration methods may not be adequate at preventing airborne exposure [177,183,184,185,186]. Various mechanisms exist by which cyanobacteria and cyanotoxins can enter the atmosphere, and these can vary depending on whether their origin is terrestrial or aquatic.

Bubbles can be generated by wave action or the result of precipitation hitting the surface of water. After forming below the surface, the outer film of bubbles can accumulate bacteria [187]. Bacterial accumulation on bubbles is dependent on bubble size, bacterial concentrations in the water, and the distance the bubble travels before reaching the surface, with larger bubbles that travel greater distances accumulating more bacteria [188,189]. As the bubble bursts upon hitting the surface of the water, bacteria concentrated on the bubble are released by propulsion of jet and film drops. Jet drops are generated from the release of water from the inside of the bubble. Film drops are composed of drops from the surface of the water upon bubble bursting. Both types of drops are capable of ejecting bacteria and potentially toxic molecules into the atmosphere [187]. For example, microcystin congeners can enter the atmosphere with the bursting of bubbles, creating lake spray aerosols (LSAs) [190,191].

Terrestrial sources of atmospheric cyanobacteria and cyanotoxins are less well understood, but may include soil, biocrusts, plant matter, and lichen [30,103,165,166,170,182,192,193,194]. Physical disruption of soil surfaces and biocrusts can disperse cyanobacteria into the atmosphere [182]. In arid regions, dust particles containing cyanobacteria and associated toxins may enter the atmosphere after being swept up by air currents moving over soil surfaces. Furthermore, up to 10^9^ bacterial cells may be present in a single gram of desert soil, suggesting that microbes will be more prevalent in the atmosphere after dust storms [169,195]. Some cyanotoxins have been detected on farmland and in groundwater [194,196], presenting the possibility of dispersal during agricultural practices. Some terrestrial *Nostoc* species form large colonies which are subject to desiccation, while others associate with fungi with forming lichen [192,193]. Desiccated or dead *Nostoc* and lichen as well as other abundant terrestrial cyanobacteria and cyanotoxins may also enter the atmosphere if they are physically disturbed.

## 4. Cyanobacteria and Cyanotoxins in the Atmosphere

Microalgae were observed as being present in the atmosphere as early as 1844 [197]. Since that time, the number of microorganisms identified in the atmosphere has greatly increased, including many cyanobacterial genera, with their presence dependent on both cellular and environmental characteristics [43,176,178,179,198]. Small cells may be more prevalent in the atmosphere according to some studies, as their small size may influence the ease with which they are swept up by air currents. Pico-cyanobacteria, which are 0.2–2 µm in diameter, are sometimes the predominant bacteria detected in aerosols [176,178]. *Microcystis* cells, which are between 3 and 4 µm in diameter, have been found during air sampling of the Baltic Sea and other habitats [199,200]. Bacterial cells of similar size have also been detected in mucosal samples (i.e., lung and nasal) from hospital patients [43], and *Microcystis* species including *Microcystis aeruginosa* were documented by Genitsaris et al. from inland samples [199]. Apart from cell size, members of the genus *Microcystis* may be more prone to aerosolization, as synthesis of gas vesicles allows them to float near the surface of aquatic environments, placing them near air currents moving across the surface of the water [21,201].

Although smaller cyanobacteria may predominate in the atmosphere, larger cyanobacteria may still be detected. Genitsaris et al. cataloged many cyanobacterial genera from atmospheric samples, which included *Lyngbya*, *Phormidium*, and *Oscillatoria* that form long filamentous trichomes [199]. *Lyngbya* species, which can reach up to 6 µm in width and commonly benthic [202], have been reported in tropical regions, but are more distinguishable in the atmosphere after harsh weather conditions such as monsoons [178,179]. Terrestrial *Nostoc* species have also been detected in aerosols [179,199]. 17 cyanobacterial species were detected in studies performed in Texas (USA) [175]. Some microalgae and cyanobacteria in this study were detected in samples taken from an airplane, implying that cell size may not be a significant factor for aerosolization and dispersal from terrestrial environments [175].

It may be assumed that greater wind speeds result in higher concentrations of cyanobacteria and cyanotoxins in the atmosphere. While bioaerosols may be positively correlated with wind speeds greater than 4 m/s, some studies suggest that wind speed may be negatively correlated with bioaerosols below 4 m/s [203,204]. Trout-Haney et al. observed a slightly negative correlation for wind speed and concentrations of aerosolized pico-cyanobacteria [176]. Wind speed may also be neutral under some circumstances, as Wood and Dietrich did not observe any correlation between wind speed and atmospheric concentrations of microcystin or nodularin [205]. Climatic factors can also influence cyanobacterial aerosolization. In India, for example, cyanobacterial genera detected in the atmosphere may change depending on the season, which is likely correlated with changes in temperature, precipitation, and humidity [179]. As mentioned above, certain genera were observed in tropical regions only after monsoons. Conditions characteristic of monsoons, such as high wind speed and precipitation, may provide the physical force necessary to aerosolize great numbers of bioparticles containing cyanobacteria [198].

Along with cyanobacteria, several cyanotoxins have been detected in air samples. Atmospheric cyanotoxins have been detected around water bodies known for harmful cyanobacterial blooms, as well as terrestrial environments. For example, microcystin and nodularin have been detected around several lakes worldwide during cyanobacterial blooms [205,206,207,208,209,210]. Anatoxin-a has been detected near a pond known for hypereutrophic conditions and harmful cyanobacterial blooms in New England [211]. BMAA has been detected in atmospheric samples around Lake Mascoma and surrounding water bodies in New England [115,198]. As various cyanotoxins have been detected in farm soil and dry lake beds such as the Great Salt Lake [171,194], inland soils may be a potential source of aerial cyanotoxins. Guanitoxin, to our knowledge, has not been detected in air samples. However, the presence of guanitoxin in desert biocrusts which may release cyanotoxins upon disruption may lend credence to guanitoxin possibly being present in the atmosphere derived from arid environments [103,166]. Limited data exists on the presence of aerosolized saxitoxin. A possible explanation is that some cyanobacteria that synthesize saxitoxin, such as *L. wollei*, are benthic, which may make entry into the atmosphere more difficult without extreme weather conditions (discussed above). Dabny did not detect saxitoxins in aerosol samples taken from a freshwater environment [212] however, other studies such as Yu et al. were able to detect saxitoxins in aerosol samples from a marine environment [213].

## 5. Health Implications of Atmospheric Cyanobacteria and Cyanotoxins

### 5.1. Possible Consequences of Exposure to Atmospheric Cyanobacteria and Associated Neurotoxins

Cyanobacteria that enter the atmosphere may present various health concerns. Cyanotoxins such as microcystin and BMAA, detected around some New Hampshire lakes, have been speculated to be causative agents of ALS cases in the surrounding area [115,214]. As mentioned above, cyanobacteria found in lung and nasal samples of hospital patients in New Hampshire were similar in size to *Microcystis* species [43]. In some cases, patients suffering from ALS were reported to live within 0.5 miles of lakes known for regular cyanobacterial blooms [214].

Cyanobacteria and cyanotoxins may also impact the health of individuals recreating on or near aquatic cyanobacterial blooms. Cheng et al. detected microcystin on air filters worn on the lapel of participants recreating near lakes with cyanobacterial blooms, implying that atmospheric microcystin was in close enough proximity to the face to be inhaled [208]. Backer et al. monitored the health of participants recreating on or near lakes with and without cyanobacterial blooms. While Backer et al. did not find a statistically significant difference in the number of participants who reported differences in health before and after recreation, many participants reported symptoms of dermal, respiratory, and digestive discomfort only after recreation, which were not experienced by participants recreating on control lakes with no visible bloom [207]. A separate study by Backer et al. showed similar results, with some participants only reporting health changes after recreation on bloom-containing lakes [206]. In both studies, microcystin was detected in atmospheric samples [206,207], supporting the idea that aerosolized microcystin may be a causative agent for the observed health changes.

### 5.2. Atmospheric Cyanobacteria and the Spread of Toxins

It should be noted that aerosolized cyanobacteria may spread hazardous substances other than cyanotoxins. Toxins such as polycyclic aromatic hydrocarbons (PAHs) and heavy metals may also be spread by phytoplankton [215,216,217], PAHs are flat planar molecules that can enter the environment through spilled fuel or biochar and are thought to be carcinogenic [218,219]. Heavy metals are present in many environments and may cause neurological complications such as ALS [220,221,222]. Studies performed on *Microcystis aeruginosa* support the hypothesis that both PAHs and heavy metals adhere to the cell surface, with some heavy metals possibly able to enhance PAH adsorption [217]. Tao et al. (2014) showed that metal salts such as copper nitrate and silver nitrate at concentrations of 500–5000 µmol/L enhanced the adsorption of PAHs such as phenanthrene onto *M. aeruginosa* cells [223]. As cyanobacteria have been detected in lung and nasal samples [43], it may be possible that exposed individuals may be inhaling multiple toxins.

### 5.3. Occupational Exposure

Occupational exposure to cyanobacteria and cyanotoxins has been reported in some instances, but documentation of exposure specifically through inhalation is limited. Stewart et al. discussed several potential instances where cyanobacterial exposure may occur in laboratory settings and mass culture facilities, with those working with dehydrated or powdered cyanobacteria possibly being more at risk [224]. Other examples included soldiers performing canoeing exercises at a UK waterbody supporting a cyanobacteria bloom, resulting in atypical pneumonia in some recruits [225,226]. As these exercises involved full body immersion in cyanobacteria contaminated water, ingestion was the most likely source of exposure, although inhalation may have also occurred, which could explain the occurrence of pneumonia in some recruits [225,226].

In certain parts of the world, significant areas of land are covered with cyanobacterial biocrusts [227]. Exposure to these crusts and cyanotoxins may result in a higher risk of developing neurological conditions. For example, unusually high rates of ALS have been observed in deployed military personnel who served in the first Persian Gulf War (Operation Desert Shield) from 1990 to 1991 versus those who received the same training and were not deployed [228,229,230,231]. While some evidence suggests that instances of ALS in active service members may be the result of administered anti-chemical warfare agents [231], contributions from cyanotoxins such as BMAA, AEG, DAB, microcystins, and guanitoxin present in desert biocrusts becoming airborne and inhaled after physical disruption cannot be discounted [103,124,166].

### 5.4. Cyanobacteria and Cyanotoxins in Indoor Environments

While little is known about how atmospheric cyanobacteria and cyanotoxins may chronically or acutely adversely affect human health, more information is available demonstrating the proximity of potentially affected individuals to airborne contaminants. Particularly concerning is that cyanobacteria are amongst the most abundant photosynthetic microbes detected in the atmosphere of indoor environments [184,185,186,215]. As many as 40 algal taxa have been detected on indoor dust particles [184]. Chu et al. found that of 26 taxa detected indoors, cyanobacteria were the most abundant, with predominant genera including those possibly originating from terrestrial environments such as *Phormidium* [184]. Some genera potentially originating from aquatic environments, including *Anabaena*, have also been detected indoors [184,186].

As cyanobacteria may be transported to indoor environments on dust particles, it may be inferred that the same is true with cyanotoxins. Since soil is a heterogenous mixture of chemically diverse materials [232], cyanotoxins could enter indoor environments via adhesion to soil-originating dust. For example, some terrestrial cyanobacteria such as *Nostoc* are capable of synthesizing multiple toxins, including microcystin [127], and it may be inferred that cyanotoxins could enter the atmosphere on dust particles from areas with high concentrations of *Nostoc*. Further sources of indoor airborne cyanobacteria and cyanotoxins include areas that use dried cyanobacteria for the preparation of food [34,52,174].

### 5.5. Dose-Response for Inhaled Cyanotoxins

Although cyanobacteria and many of their associated toxins negatively impact the nervous system, the mode by which exposure occurs may influence the severity of symptoms. While data showing dose–response relationships for inhalation is not available for all classes of cyanotoxins discussed in this review, some cyanotoxins such as MC-LR, anatoxin-a, and BMAA have been derived. LD_50_ concentrations for MC-LR and anatoxin-a decrease upon exposure via nasal administration as opposed to other routes such as gastric intubation, reflecting the potency of direct inhalation [233,234]. Inhalation-based exposure to MC-LR resulted in increased liver size which is indicative of liver damage and increased proportionally to exposure concentrations [233]. Simultaneous exposure to MC-LR and anatoxin-a via inhalation can also result in synergistic toxicity which becomes more apparent with increasing toxin concentrations. However, it should be noted that mice exposed to aerosolized MC-LR did not show any adverse health defects, although this could be explained by technical limitations and the inability of mice to breathe large enough quantities of aerosolized toxins to exhibit symptoms of intoxication [233]. Although not administered via inhalation, Fawell et al. showed that mice exposed to anatoxin-a displayed dose-dependent changes in respiratory action at sublethal doses [235]. Benson et al. also showed a dose-dependent response in mice exposed to 200–265 µg/m^3^ of MC-LR, with exposed mice inhaling MC-LR for a period of seven days. Mice inhaling MC-LR for 0.5, 1, and 2 h displayed concurrent increases in the instances of nasal lesions and nasal epithelium necrosis, with both higher concentrations and longer periods of exposure [236].

BMAA has been shown to damage cells of the olfactory bulb and olfactory tract [42], but the degree to which BMAA may be toxic upon inhalation may be organism dependent. For example, Scott et al. showed that rats administered BMAA via inhalation did not exhibit any adverse health effects upon exposure to environmentally relevant concentrations. As concentrations of neurotoxic degradation products of BMAA such as 2,3-diaminoproprionic acid (DAP) increased proportionally to concentrations of inhaled BMAA, this implied that rats may show a degree of resistance to the toxic effects of inhaled BMAA [237]. However, Pierezan et al. showed that mice exposed intranasally to BMAA displayed selective damage to the olfactory bulb, as well as decreased viability and neurite growth in primary cultures of mice olfactory cells. It should be noted that Pierezan et al. did not observe adverse effects of BMAA at concentrations below 100µM in olfactory bulb neurons. Mixed cultures of olfactory neurons and glial cells showed decreased viability at 500µM concentrations, but not at 250µM, demonstrating the critical nature of BMAA dose on inhalation toxicity [238]. See Table 2 for a summary of the results discussed above.

### 5.6. Biomarkers for Inhalation of Cyanotoxins

Studies examining biomarkers for exposure to cyanotoxins, specifically through inhalation, are somewhat lacking, although it could be assumed that the biomarkers mentioned above for oxidative stress, neuroinflammation, and protein aggregation could be seen in cases of inhalation toxicity. One study reported nasal legions in mice exposed to MC-LR for seven days, in addition to increased expression of two unidentified proteins in mouse plasma which directly correlated with MC-LR exposure [236]. Although not conducted in the context of inhalation, changes in gene expression and the phosphorylation of enzymes involved in cell division have been documented in human-airway epithelial cells upon exposure to sublethal concentrations of cylindrospermopsin [239,240].

## 6. Mitigation of Cyanobacteria and Cyanotoxins in the Atmosphere

Several strategies exist which may lessen the impacts of harmful cyanobacterial blooms. However, few studies have been conducted with the intention of reducing cyanobacteria and cyanotoxins in the atmosphere. Since many cyanobacteria originate from aquatic environments, strategies aimed at reducing aquatic blooms may also reduce the amount of atmospheric cyanobacteria and cyanotoxins.

Current control methods utilize chemical agents which induce flocculation, the aggregation of dispersed bacterial cells in solution, forming flakes known as flocs [241]. Chemical agents such as aluminum or iron-based compounds and organic compounds like chitosan flocculate cyanobacteria by neutralizing the cell surface and precipitating them from solution [28,242,243,244]. A method known as Floc and Sink utilizes clay particles which act as ballast, causing the flocculated cyanobacteria to sink and removing them from the surface of waterbodies [28,245]. Although blooms may recover, temporarily removing cyanobacteria from surface waters may reduce aerosolized cyanobacteria, as fewer cells would be released into the atmosphere if only for a limited period. Flocculated cells may also not adhere to bubble films, possibly reducing the number of cells available to enter the atmosphere through mechanisms such as bubble bursting [187].

Reducing the amount of available nutrients in an aquatic environment may also reduce concentrations of cyanobacteria and thus cyanotoxins. To reduce bioavailable nutrients such as phosphates that enter aquatic environments as agricultural runoff, mitigation strategies include reducing fertilizer use on watershed farmland [245,246,247]. For example, cover crops such as tillage radishes grow extensive root systems capable of drawing nutrients upward from deeper soil layers to be utilized by cash crops, and could possibly reduce the total amount of phosphorus containing fertilizers needed for application to farmland [245,248,249,250]. Strategies that mitigate phosphates already present in waterbodies include the addition of compounds such as alum, clays, and lanthanum-modified bentonite, all of which precipitate phosphate anions from surface waters [28,251]. In either case, this limits bioavailable phosphorus and may reduce the growth of aquatic cyanobacteria, and the subsequent number of cells available to be liberated into the atmosphere.

Nie et al. investigated the presence of cyanobacteria on HVAC filters and found cyanobacteria of the genera *Pseudanabaena*, *Nodularia*, and *Letpolyngbya* [252]. These findings suggest that air-filtration methods may reduce concentrations of airborne cyanobacteria and lessen human exposure to cyanobacteria. Gaston et al. employed a filtration system in which a combination of high-efficiency particular air (HEPA) filters, air conditioning (AC) filter cassettes, and face mask pieces were used to filter airborne cyanobacteria. Roughly 80% of aerosolized cyanobacteria detected in this study were prevented from entering the atmosphere and were attached to one or more components of the filtration system [253], suggesting that in-house air filters along with personal protection face masks may help to minimize human exposure to airborne cyanobacteria.

## 7. Research Gaps

Although much is known about exposure to cyanobacterial toxins via drinking and recreational water as well as from contaminated crops and food, the contribution of the airborne cyanotoxin exposure route to toxicity is largely unknown. A further compounding factor is that, unlike exposure via water, avoiding toxic compounds within air is more difficult due to the necessity of inhalation and penetration of cyanobacteria into indoor environments.

If such exposure is unavoidable, current research gaps include accurate and simple means to detect and quantify cyanobacteria and cyanotoxins in air to provide an early warning system, like that provided by US national weather services for brevetoxins and wildfire smoke [254,255,256]. Such a system may be particularly important for large lakes, such as Lake Erie and the Great Salt Lake, or marine environments. Regarding Lake Erie, persistent cyanobacterial blooms, largely comprising the genus *Microcystis*, have the potential to cover large surface areas with microcystin contamination [12,257]. With smaller lakes, it may be sufficient to simply use the “precautionary principle” and post warning notices when toxic blooms are present or likely to be airborne. Although this may be sufficient for members of the public periodically recreating on lakes, individuals whose residences are near such waterbodies may be at increased risk of acute and chronic adverse health effects [214].

Other research gaps include the potential to filter air samples and remove cyanobacteria and their components as a preventative measure. Although some experiments have been performed in laboratory settings [253], publicly available technologies to specifically serve this purpose have, to our knowledge, not been made available. Non-specifically, such remedies may take the form of simple HEPA filters or bespoke filtration systems. It is likely that most airborne exposure occurs through the inhalation of water droplets, cellular material, and debris or dust particles, as shown by the presence of cyanobacteria in human lung samples and indoor environments [43,184,185,186]. Furthermore, the aerial exposure route may manifest in different toxicological outcomes if compounds are inhaled. This is largely because compounds may enter the body through the olfactory bulb and subsequently have the potential to bypass the BBB, resulting in neurotoxicity. BMAA, for example, was present in mouse olfactory bulbs following direct unilateral intranasal instillation, providing evidence that BMAA can bypass the BBB and enter the brain directly through the olfactory bulb [238]. Cyanotoxins such as BMAA, DAB, and AEG have also been detected in the olfactory bulbs of postmortem human subjects, which corresponded with the presence of proinflammatory proteins such as IL-6 and CASP1 in subjects with advanced Alzheimer’s Disease. Concurrently, the olfactory tracts of postmortem Alzheimer’s Disease patients showed pathologies such as neuropil vacuolation, gliosis, and the presence of reactive microglia and tauopathies [42].

Globally, the presence and persistence of cyanobacterial blooms is highly variable, with some lakes having large persistent blooms (e.g., Lake Erie), some serving as permanent cyanobacterial ecosystems (e.g., Rift Valley Lakes, Great Salt Lake), and the affected majority being small lakes with periodic/annual cyanobacterial blooms [38,258,259,260,261,262]. Therefore, a greater understanding of temporal and geographic ranges of blooms may aid in the identification of at-risk human populations. This includes the need to obtain accurate and rapid determinations of toxin concentrations to ascertain potential degrees of exposure. This can be easily achieved using low-cost, low-tech detection devices such as immunoassays, as evidenced by self-administered COVID-19 tests during the pandemic [263]. Furthermore, if properly calibrated, techniques such as qPCR may also be amenable to this task to increase the amount of available data for assessing disease patterns and trends in populations [264,265,266].

Although cases of acute toxicity from cyanobacterial toxin exposure are relatively easy to determine and investigate, understanding potential chronic effects requires greater effort. Under such scenarios, as a person may be exposed to cyanobacteria and their toxins at multiple times over multiple years, a better understanding of the amount and frequency of exposure would be invaluable [267,268,269]. Although this would require many analyses to be performed, the data may be amenable to the application of techniques such as machine learning to look for patterns in exposure without human bias or interference [267,268,269]. Another research gap with respect to chronic exposure concerns genetic differences within human populations which may affect susceptibility to cyanotoxin exposure. With some neurodegenerative diseases such as ALS and PD, a percentage of cases are thought to be caused by genetic predisposition with environmental triggers. By applying health assessment criteria such as those devised by Bradford-Hill, a better understanding of the ability of cyanobacteria and their toxins to cause adverse human health effects and disease can be achieved [270,271].

The potential list of diseases associated with exposure to cyanobacteria and their toxins continues to grow [52,267,268,269,271,272]. With the correct tools and surveillance networks in place, a better understanding of less-studied exposure routes can be achieved. The increasing analysis of cyanobacteria and their toxins in air indicates that this exposure route may be more important than previously considered. Having a better grasp on the occurrence and prevalence of cyanobacterial toxins in air may also lead to a more informed risk assessment. This may include a better understanding and partitioning of toxin allocation amounts for health protection and may also result in changes to recommended permissible cyanotoxin concentrations in water, food, and other compartments.

Perhaps the greatest research gap concerns the human exposome and the totality of exposure. Whether by water, air, or food, humans are exposed to a variety of compounds with potential acute and chronic deleterious effects. A better understanding of the variety of compounds that people are exposed to is required, along with data and research concerning potential synergistic toxicological effects [273]. To protect human health from the actions of cyanobacteria and cyanotoxins, the quantity of these compounds and how they interact in human bodies will provide the means to better protect human health. This is especially pertinent when combined with likely adverse effects of climate change on harmful cyanobacterial bloom occurrence and distribution.

## Figures and Tables

**Table 1:** Cyanotoxins and neurological conditions.

Cyanotoxin	Oxidative Stress	Neuro-Inflammation	Protein Misfolding	Neuro-Stimulation	Neuro-Inhibition	References
Anatoxin-a	+	-	-	+	-	[44,45,46,47]
Guanitoxin	+	-	-	+	-	[48,49]
Saxitoxin	+	+	-	-	+	[50,51,52,53,54,55]
Amino Acids *	+	+	+	+	-	[56,57,58,59,60,61,62,63,64]
Microcystin	+	+	-	+	+	[65,66,67,68,69,70,71,72,73,74,75,76,77]
Cylindrospermopsin	+	+	-	-	+	[78,79,80,81,82,83,84,85,86,87,88,89,90]

* Includes BMAA and isomers.

**Table 2:** Toxicological analysis of inhaled anatoxin-a, MC-LR, and BMAA in rodent models.

Cyanotoxin	Route	LD_50_	Test Organism	Reference
Anatoxin-a	Inhalation	2000 µg/kg	Mouse	[233]
Microcystin-LR	Inhalation	250 µg/kg	Mouse	[233]
Microcystin-LR	Aerosol	NA *	Mouse	[233]
Microcystin-LR	Inhalation	43 µg/kg	Mouse	[234]
BMAA	Inhalation	NA	Rat	[236]

* This study was limited to aerosol exposure at 0.0005 µg/kg. No deaths were observed at 0.0005 µg/kg.
